# Dentistry as a "Fine Art."

**Published:** 1867-06

**Authors:** Norman W. Kingsley

**Affiliations:** Professor of Dental Art and Mechanism, in the New York College of Dentistry.


### ARTICLE V.

### *Dentistry as a "Fine Art."*

By Norman W. Kingsley, Professor of Dental Art and Mechanism, in the New York College of Dentistry.

That "Dentistry is a Science and an Art" is a statement that at this day it seems hardly necessary to reiterate. The phrase has a royal sound, and its frequent repetition, shows that it is a favorite expression ; and yet among the multitudes who earn their livelihood by practicing dentistry, how few know the full meaning of the words, and how little there is in their practice to justify the assertion.

To judge by its fruits, how much more there is of Empiricism than of Science ; and how much more of rude and bungling Mechanism than of Art. Nevertheless, Dentistry *is* a Science and an Art, and the researches of the past four years alone, together with the contributions to its literature, give a legitimate claim to the first part of the proposition. Months and years have been spent in the prosecution of it as a Science, and volumes record its results ; but as an Art, capable of taking rank as one of the

Fine Arts, Dentistry has rarely, if ever, found in our Journals, an advocate; has rarely found else than a flippant consignment to the workshop, where the very idea of art comprehends only ordinary mechanics.

As a consequence, Artistic Dentistry has never risen, except in rare individual cases, to anything above mechanical dentistry; the very term by which the department is known is often used as one of reproach, and the productions of these dental mechanics are a standing disgrace. In every assemblage, public or private, on the street, in the drawing-room or wherever we may turn, we see displayed their hideous deformities. It becomes a serious question whether the art of dentistry, aside from operations upon the natural teeth, has, with all the inventions and "improvements" of the last decade, advanced one iota.

The Operative department has assumed to be the department, *par excellence* and *per se*, and we see the results in the education of the new professional generation, who ignore as unworthy *their* exalted talents any knowledge of mechanical dentistry, not realizing that a mastery of all its elements will do more to educate and qualify them for perfection, even in the one department, than any other course that could be pursued. We believe that it can be demonstrated beyond a peradventure, that the ignored and despised branches of dental practice can lay a well grounded claim to be considered as a Fine Art, capable of the highest idealization, ranking side by side with Poetry, Music, Painting and Sculpture, capable of appealing, though in a more limited manner to the same sentiments and emotions, and requiring for their expression the identical talent and same imagination which characterise the votaries of her predecessors.

With the ancient Greeks, all works which exhibited skill were called works of Art, and to the present day the term Art in its broad signification is applied to every skillful, physical or intellectual performance, from the making of a shoe to the modeling of a statue, from the pantomime of the stage to the oratory of the forum. But as the Arts have multiplied, terms of distinction have become necessary; as, Fine Arts and Mechanic Arts with all their subdivisions.

The distinguishing characteristic of the fine arts is their ideality, in this the line of demarcation between them and the mechanic, and all other arts is unmistakably distinct.

It is for this feature we look in any work that claims this high rank, and by this standard we judge of its pretension. The mechanic arts are distinguished for their physical utility; they may demand consummate skill for their execution, they may require for their development rare inventive faculties, and their combinations of mechanical principles may be truly wonderful, but their individual works require but little effort of the brain in their re-production; education in skillful manual labor without the capacity to originate a single new idea is all that is required. The laws which govern their re-production are those of mathematics, and to be able to copy a given form with exactness is the sum of the talent required.

They may be directly of more practical value to mankind, but they can make no appeal to the finer emotions of our being. In all that excites the imagination, that calls into action the affections or leads the mind away from the contemplation of the material and sensual, they are dumb.

In like manner the feats of the acrobat and juggler excite our wonder and admiration, but like the true mechanic arts have not an element of ideality in them.

The ideal or Fine Arts therefore may include Poetry, Music, Painting and Sculpture. These require for their development the possession and exercise of the same mental faculties, are governed by the same general rules, and have one common ultimate object.

Poetry, of all the arts, stands deservedly at the head, because the most subtle, and at the same time the most potent in its influence. It is the least material of all, the farthest removed from sensible objects ; it has the greatest scope, admitting the treatment of all subjects, and its power over the imagination is the most complete. "In regard to the objects of the visible world one cannot conceive a greater distance between what it depicts, and the manner of depicting. By the combination of words alone used as language, poetry gives full expression to every idea from the most powerful to the most delicate, and presents scenes so vividly as well as so variedly to the mind of the reader that the impressions are both pleasing and permanent."

Music takes precedence over Painting and Sculpture in the order of the Fine Arts ; its claim to superiority being based upon inherent qualities of human nature ; by whose control all that appeals to our emotions is more regarded than what appeals to our understanding. Objects of sight, while they please the eye by their color, form an arrangement, affect mainly the mind, and only indirectly approach the heart.

But music speaks directly to the soul. Its notes are the re-production and the refinement of natural tones of pleasure, anger, fear, distress, etc. These tones, music cultivates, and by prolonging and combining them, expresses feelings and awakens every variety of emotion into sympathetic activity. Thus the tender pipings of the flute call forth the love of enjoyment ; the clear bugle notes find responsive echoes in the hearts of mountaineers and huntsmen ; the orchestral overtures send thrills and throes through fancy loving souls ; and the choruses of Mendelsohn and Mozart fill man's spirit with ecstacy of joy or wonder, or with solemn awe.

More than any other Art, is music universal. A language without words, music addresses the feelings by tones, everywhere understood and unmistakable ; and it leads forth the emotions to an enjoyment which words cannot give, nor can even express.

Painting in the order of the ideal Arts holds the third rank. It is more limited in its scope than either of its predecessors, and expresses its sentiments or tells its story by the color and lineal appearance of bodies.

But while it cannot undertake the relation of a succession of events in one representation, it has the advantage over Poetry, in that its language is universal, easily recognized, and needs no interpreter or translator of its meaning,

"A true picture tells its own story,"

and in no way does the common mind receive more vivid and lasting impressions of portrayed events, than by the productions of this art.

Many pictures there are whose sentiment is sacrificed to that which merely pleases the eye, the gratification of the senses by the harmony of forms and color, being the highest aim of the artist. It is this degeneracy into mere physical representation, and the limited nature of its power, that places it as an ideal Art in the rank that it occupies.

The art of sculpture while requiring for its execution the very highest order of mental faculties, is generally placed in the scale of ideal arts, below that of painting, because represented solely by form, and without the aid of color, it is more limited in its subjects and more material in its exhibitions.

In the selection of the human figure, the most perfect of all forms, it finds its grandest achievements in depicting all gradations of intelligence, affection, sentiment, action or passion: sublime, heroic or tender, and in all orders of beings from the exalted supernatural to the lower gradations bordering on the brutes. Although the most limited in its scope, it is not so liable to degenerancy as its sister art of painting; to be suceessful, its delineations must be above that of simply copying nature, and its power over the beholder is often greater than any other art could give to the same subject.

It is the most enduring of all arts; the material chosen for its medium being the most independent of all the mutations of time. Of the peoples of the by-gone ages, the only records left to us that give even a passing glimpse of their existence, are their sculptured monuments.

Like painting and music its language is universal, and like painting when it ceases to appeal to the imagination, and seeks only to please the senses by the beauty of form, it degrades its character and fails of its true mission.

"Nothing brings people of other nations so vividly before us as their works of art. They tell us of their religion, of their social dwellings and customs, of their advance in civilization and religious culture. To the study of ancient history a knowledge of the arts as practiced by different nations is indispensable. Language is more or less subject to change and decay, and the significance of many expressions is lost to those who do not use it as a vernacular tongue." Many nations have existed with no written language, but the Almighty in blotting them out from the face of the earth has permitted their works of art to live, from which something may be learned of their rise, progress and character.

Architecture is very commonly regarded as one of the fine arts, and ranked next to sculpture and painting. Modern architecture is addressed to the eye and the intellect alone, and not to the imagination, and the ideal character necessary to distinguish a fine from a mechanic art is wholly wanting—wherever this element does exist in buildings of the present day, it has borrowed it from sculpture. But architecture among the ancients was one of the earliest of symbolic languages. The pattern shown to Moses in the Mount by God, as the model for the building of the tabernacle, embodied the very highest order of ideal art.

Every post, every board and every bar, every ring, and every curtain, were typical of man's redemption from sin, and spoke a language unmistakable, and clearly comprehended by the Jews. And so with every monument of the ancient heathen connected with their religion, every temple, every idol and every altar appealed to the imagination and spoke an ideal language.

Architecture was then emphatically a "fine Art," but at the present day it is but an imitation of the dead past. The powers that called it into existence are gone, and the emotions to which it gave birth have died out. We imitate its corporeal form, but the spirit that gave it life is forever departed.

We have been thus specific in our description of the Fine Arts for a more thorough understanding of grounds upon which we shall base the claim of Dentistry to be ranked with them. No performance of the dentist can make any pretension to be a fine art, separate and distinct from all others; but as a sub-division or speciality of one of the arts, dentistry is entitled to a consideration which it has never received. We shall endeavor to show this alliance, and prove that, so far as its scope will allow, it is governed by the same general rules which control its allied art.

Dental practice, by an inherent law and by common consent is divided in the main into two departments; the one commonly termed the "Operative" which is made to include all efforts for the preservation of the natural teeth, and all surgical operations in the buccal cavity; the other called "Mechanical" which includes the making of all appliances for the correction of deformities of the buccal cavity; but principally the making and inserting of Artificial Teeth.

In the practice of Operative Dentistry as has been before intimated, there has grown up an unwarrantable assumption that all that was refined and cultivated, all that was worthy the exercise of our noblest faculties in the pursuit of our profession was to be found in this department—and mere mechanics, wholly unqualified by education in Science and Art, were deemed capable of practicing the other.

The only performance of the Operative dentist which requires a talent and skill equal to the mechanic arts is the introduction of fillings into the cavities of decay, and this skill is mere manual dexterity guided by good judgment; its highest achievements at the present day are in the so called contour fillings made of gold, in which an attempt is made to restore the form of a tooth injured by accident or decay.

Contour filling when carried to its highest state of perfection in restoring the actual or the typal form of the lost organ, can present no stronger argument to be considered an artistic performance than that of a copy or an imitation. If a copy it is purely mechanical, if an imitation of the typal it may lay a feint claim to ideality. This is true when carried to its ultimate; but practically nine-tenths of what are called contour fillings are not entitled to any such distinction. Nuggets of gold they are, built on to deformed teeth, carried in many instances far beyond the borders of decay; lapping over and building up on solid enamel to a general level, obliterating all inequalities and all character, and failing most completely to illustrate the possession of any other talent than the skillful manipulation of gold, excellent advertisements of the craft they undoubtedly are, but are certainly of very questionable taste.

Every tooth has an individual character and expression, not only in harmony with every other in the same mouth, but by the same divine law, in harmony with the features and the character of the creature, be he animal or man.

These physical characteristics are so marked and prominent that the merest novice has no difficulty as a rule in locating any human tooth that has been removed from its fellows; and yet of the attempts at restoration of any large portion of the crowns of teeth by dentists, how few there are that bear any very close resemblance to the original form of the lost part.

If a cast were taken of the restoration and examined separately, how few would identify it as being any portion of any tooth. The cusps, the depressions, the sutures, the easy and graceful outlines and all that marks the individual tooth are wanting.

With the same portion of a natural tooth even duplicated in another material, as a perfect copy in plaster, there would be no hesitation in identifying its locality with a tolerable certainty, but a cast taken of many a restoration would not be suspected of its original application.

Of the utility of this branch of practice and as a field for scientific research—pecuniarily remunerative—there can be no question, but as affording an opportunity for esthetic culture, it bears no comparison with its associated department.

If Sculpture necessarily ranks below Painting in the scale of the fine Arts, because more limited in its range, and Painting for the same reason below Poetry ; we must therefore place all operations on the natural teeth, as artistic performances in rank, below that of the substitution of artificial ones.

As an Art it is but a department of Sculpture. *Form* in individual members, *form* in grouping and arrangement, and *form* as a medium of expression are equally the distinguishing characteristics of both Sculpture and Dentistry.

(To be continued.)

